# Common Pitfalls in Management of Inflammatory Bowel Disease

**DOI:** 10.4021/gr2009.07.1305

**Published:** 2009-07-20

**Authors:** Lakshmi Pasumarthy, James Srour, Cuckoo Choudhary

**Affiliations:** a York Hospital, Dept of Medicine, 1001 S. George Street, York, PA 17405, USA; b Thomas Jefferson University Hospital, Philadelphia, PA, USA

**Keywords:** Inflammatory bowel disease, Ulcerative colitis, Crohn’s disease, Tumor necrosis factor, Azathioprine, 6-mercaptopurine

## Abstract

Our understanding of inflammatory bowel disease (IBD), treatment options, complications and their management has expanded significantly over the past few decades. When caring for patients it is important to remember the complexities of pathogenesis and pharmacology. This review is to identify errors in diagnosis, treatment, complications and preventive care issues that arise while caring for patients with IBD and to provide recommendations and information that can be shared with patients and their health care providers. A review of the literature was undertaken using MEDLINE from 1981 to present. We included randomized controlled studies, case-control studies, and review articles. There are many associated conditions and complications recognized in patients with IBD and current treatment strategies do result in many side effects, some are serious and some are not widely recognized. With the advent of anti-TNF therapies and the newer 5-amino salicylate derivatives, options available have increased significantly. It is also important to remember that these patients are followed by more than one health care provider and it is important for all involved to communicate the plan of action.

## Introduction

As many as 1.4 million persons in the United States and 2.2 million persons in Europe suffer from inflammatory bowel disease (IBD). The strongest environmental factors identified are cigarette smoking and appendectomy, both being risk factors for Crohn’s disease (CD), protective factors for ulcerative colitis (UC) [1].

We will outline the errors made in the management of the condition as well as the complications that arise from the condition and its treatment. We have reviewed the diagnostic and therapeutic modailties already approved and accepted. Though there many newer medications and agents being investigated, these are not within the scope of our discussion.

## Diagnosis

### Serology

Diagnosis of IBD is made after a thorough history and physical, endoscopy, reviewing the histology since successful treatment is achieved through correct diagnosis. We would like to comment on the difficulty encountered with serologies. The diagnostic gain of a test is minimal when the pre-test probability of disease is very high or very low, and the gain from diagnostic testing is maximal when the pre-test probability of disease is somewhere in the middle. The same can be said of the serological assays for IBD. The most commonly used antibodies are deoxyribonuclease (DNase)-sensitive perinuclear antineutrophil cytoplasmic antibody (DNase-sensitive pANCA), IgA and IgG antibodies to Saccharomyces cerevisiae (IgA and IgG ASCA), and antibody to Escherichia coli outer membrane porin (anti-OmpC). Serological assays should play a limited role in the diagnosis of IBD. These serological assays may be helpful when the results of the appropriate evaluation are inconclusive [2]. Although the test characteristics in regards to sensitivity and specificity are reasonably good for the most comprehensive serologic panel for IBD, the serologic tests should not be considered a diagnostic tool, and treatment for IBD should not be initiated solely on the results of serologic testing [3].

As pointed out by Dubinsky, the titers may also be used for prognostication in certain cases. The presence of certain markers, such as ASCA, is an indication for a high risk of postsurgi­cal CD or fistulization of the pouch, and the presence of multiple antibodies indicates more aggressive disease course.

### Endoscopy

Although conventionally it is accepted that rectal sparing or patchy involvement should increase suspicions of CD, there are circumstances where patchiness can be observed in UC. This may be seen in patients who have received prior local or systemic therapy. Rectal sparing usually occurs if the patient has applied topical enemas [4]. “Skip lesions” seen on colonoscopy may be secondary to partial healing in ulcerative colitis. “Cobblestoning” is a result of ulceration and healing and is seen in both UC and CD. Therefore, it is important to avoid placing too much emphasis on the traditionally described findings.

Capsule endoscopy is useful in bleeding and small bowel assessment, but poses a few problems in patients with CD, potential for capsule getting stuck, frequency of nonspecific aphthous ulcers in patients taking NSAIDs. MR enterography and CT enterography have been cited as imaging modalities with comparable efficacy.

Patients initially diagnosed with either UC or CD may demonstrate with time additional features which may support or be against the initial diagnosis. In a study from Norway out of the 527 patients initially diagnosed with UC, 88% had their diagnosis confirmed on follow up in 1-2 years. 91% of 228 patients with CD had their diagnosis confirmed in the same follow-up period. 36 patients were diagnosed originally to have Indeterminate Colitis. On follow- up, 33% of these were re-classified as UC and 17% as CD. The study illustrates the importance of the re-evaluation of the initial diagnosis as up to 10%, both among patients with UC and CD, were reclassified at follow up [5]. This fact was also demonstrated by Langevin et al in a study involving 96 patients with an initial diagnosis of ulcerative proctitis, 14% of them developed features of CD in 29 months of follow-up [6]. Given this finding, it is not unreasonable that a patient be given a provisional diagnosis with the caveat that it may change and the clinician should follow up and assess for accuracy of initial diagnosis.

It is also important to avoid confusion with other conditions which can cause similar symptoms and endoscopy findings. The endoscopic appearances of the mucosa and the histologic changes in infective and inflammatory colitis may be virtually indistinguishable [7]. A third of patients presenting with mucoid bloody diarrhea and suspected IBD have an infective etiology. Patients with IBD have the propensity for bacterial superinfection [8]. The most common enteric pathogens implicated are Campylobacter, Salmonella, Shigella, Amoeba and Clostridium difficile. In the majority of cases the history, presentation in addition to serological tests and stool cultures help in the differentiation between infective colitides and IBD. Typical examples of other conditions that mimic IBD are ischemic colitis, diverticular colitis.

## Medical therapy

Table 1 is a list of medications approved for the treatment of IBD.

**Table 1:** Medications approved for treatment of IBD

Class	Examples	Indications
Sulfasalazine and 5-amino salicylates	Azulfidine-Olsalazine, Asacol, Pentasa, Balsalazide	Mild to moderate UC and CD
Corticosteroids	Hydrocortisone, Prednisone, Budesonide	UC and CD
Immunosuppressives	Azathioprine, 6-Mercaptopurine, Methotrexate	Evidence for CD > UC. MTX-no role in UC
Anti-TNFα Antibody	Infliximab, Adalimumab, Certolizumab pegol	Severe UC (Infliximab)/ all 3 for CD
Antibiotics	Metronidazole, Trimethoprim-sulfamethoxazole, Ciprofloxacin, Clarithromycin,	Ancillary in treatment of IBD

### Glucocorticoids

#### Topical

Mild cases, especially when only the distal colon is involved, respond well to topical therapy with or without adjunctive oral therapy [9]. Non-systemic steroids such as oral and rectal budesonide for ileal and right-sided CD and distal UC respectively are also effective in mild-moderate disease [10]. Treatment with budesonide enema in active distal ulcerative colitis was comparable to treatment with conventional prednisolone enema. A prolongation of the treatment time from 4 to 8 weeks doubled the clinical remission rate in both groups. However, budesonide may be preferable to prednisolone since it causes less systemic effects as reflected by a lack of plasma cortisol suppression.

#### Oral

In cases with CD, mild to moderate ileal disease responds well to oral Budesonide [11]. For mild to moderate UC, a dose-response effect for prednisone 20-60 mg/d has been reported, but doses greater than 60 mg/d confer no additional benefit [12].

In patients with severe UC, there is a concern for relapse when using a low dose of prednisone. Similarly using very high doses or for a prolonged period exposes the patient to avoidable side effects which span nearly all the organ systems. If the patient is treated several times with glucocorticoids, even if topical, physicians must start monitoring them for side effects such as glucose intolerance, gastric irritation, cataracts, increased risk of fracture [13].

In patients with severe UC, it is important to use doses of glucocorticoids equivalent to hydrocortisone 100 mg intravenous every 8 hours. If there is no response in 3-7 days, consider steroid resistance, since the management depends on quick diagnosis and carefully ruling out other confounding conditions [14]. Involving surgery early on is very important in order to avoid colonic perforation which in some cases can prove to be fatal.

It may also be appropriate to perform flexible sigmoidoscopy to rule out opportunistic infections such as cytomegalovirus (CMV) colitis [15]. If CMV colitis is ruled out and the patient is no better, consider treatment with intravenous cyclosporine/ infliximab/ or surgery.

### Sulfasalazine and 5-amino salicylates

Rectal 5-ASA has been found to be superior to steroid enemas in the management of distal ulcerative colitis [16]. Oral 5-ASA compounds, including olsalazine, sulfasalazine mesalamine, balsalazide are effective in inducing remissions in active ulcerative colitis although side effects are significantly higher with sulfasalazine [17, 18]. Sulfasalazine is effective for the treatment of Crohn's colitis, but is less useful in patients with active ileitis. This diminished response probably reflects the need for colonic bacteria to cleave the drug to release the active 5-ASA moiety [19].

If a little is good, more is better. There is evidence for dose dependence in 5-ASA therapy for oral preparations, with optimal effects being observed at 4.8g/ day of mesalamine as compared with 2.4 g/day.The latter works well in mild cases while higher doses are utilized in moderate/extensive disease. Both doses of mesalamine had similar saftey profiles and both were well tolerated [20, 21].

Adequate attention should be paid to patients’ level of compliance with the prescribed medicine regime. Nonadherence with medication increases the risk of clinical relapse among patients with quiescent ulcerative colitis. In a study by Kane et al, it was found that patients who were not adherent with medications had more than a fivefold greater risk of recurrence than compliant patients (hazard ratio = 5.5; 95% confidence interval: 2.3 to 13; p < 0.001) [22].

### Immunomodulator therapy

6-Mercaptopurine (6-MP) and its prodrug azathioprine (AZA) are thiopurine analogues and are immuno-modulatory agents. Of the AZA compound, 88% is converted via nonenzymatic process to 6-MP. AZA or 6-MP should be considered in UC patients who are refractory to maximal doses of 5-ASA medications and require oral corticosteroids to control symptoms. Both agents are used as “steroid sparing” medicines. As a general rule, consider using these drugs in patients who require four or more months of continuous corticosteroid therapy to control symptoms and/or three or more flare-ups in a given year that require steroids to achieve remission.

In the pediatric study by Markowitz and colleagues, only 4% of patients of the 6-MP group required another course of steroids within 540 days after being weaned off of prednisone, clearly in contrast to the 57% of pediatric CD patients receiving placebo with a need to restart prednisone within 360 days (P < 0.0001) [23].

AZA and 6-MP are also effective for maintaining remission for many years in patients with CD whose remission was initially achieved with these drugs [24]. There is also expanding evidence that AZA is effective as a post-operative maintenance therapy [25]. The risk of infection with these medication ranges between 0.3%-7.4% and includes herpes viruses, human papilloma virus and upper respiratory infections [26].

Increased risk of hematologic malignancies has also been associated with prolonged leucopenia in IBD patients on 6-MP, and EBV-positive lymphomas have also been found more frequently in patients exposed to 6-MP or AZA [27, 28].

One in 300 individuals lacks Thiopurine methyltransferase (TPMT) and 11% of the population is heterzygous with intermediate activity. This enzyme is essential for metabolism of the purine analogs. It is important to check TPMT enzyme activity and monitor the CBC monthly to avoid severe myelosuppression. Patients with low enzyme levels are at risk but can be treated with careful dosing titration. Patients with absent enzyme should not be treated with these drugs [29]. It is important to follow up on regular bloodwork monitoring (full blood count, liver panel) after starting antimetabolites and judiciously use these in patients not responding to adequate weight based dosing.

### Monoclonal antibodies

The monoclonal antibodies currently being used in IBD are infliximab, adalimumab, certolizumab pegol. These agents mediate pro-inflammatory processes central to the pathogenesis of IBD. Adalimumab and certolizumab pegol have an advantage of subcutaneous administration rather than intravenous administration required for infliximab. The safety record with infliximab and adalimumab is longer than with certolizumab pegol. Only infliximab is approved for UC. All three agents are approved for CD. We are not discussing Natalizumab in our paper.

Patients with moderate-to-severe active ulcerative colitis treated with infliximab were more likely to have a clinical response than were those receiving placebo as shown in two randomized placebo controlled trials in ACT1 and ACT2 [30]. Patients treated with moderate to severe Crohn’s disease treated with infliximab also had significantly higher remission rates as demonstarated by the landmark ACCENT I and ACCENT II trials.

The preliminary data from a comparison of top-down versus step-up therapy were presented in which combined immunosuppression was compared with a conventional step-up approach in 129 CD patients with a CD activity index of at least 200[31]. Patients had to be steroid nave and had not been exposed previously to infliximab or antimetabolites. The step-up algorithm included induction of remission with corticosteroids, followed by repeat courses of steroids and azathioprine in the case of new exacerbations, and eventually infliximab if these therapeutic interventions failed. Mucosal healing was found in 73% of patients assigned to top-down treatment versus 30% in the conventional group at 24 months.

The FDA published a summary of 47 cases of HF associated with infliximab reported to the Adverse Events Response System (AERS) through January, 2002. In a patient who develops heart failure while on a TNF inhibitor, a drug-induced cause should be suspected and the medication should be discontinued. TNF inhibitors have rarely been associated with the development or exacerbation of neurologic disorders associated with demyelination, such as multiple sclerosis. However, the true nature of this association has not been established. Anti-TNF therapy should be discontinued immediately in any patient with suspected demyelination.

Before starting monoclonal antibodies it is important to screen for tuberculosis with PPD testing and chest X-ray, understanding that anergy is common in IBD. It should be stressed that patients are exposed several opportunistic infections including pulmonary and systemic fungal infections, and that their immune defense mecchanisms are compromised. There is also a concern for exacerbation of latent infections such as hepatitis B and increased occurrence of non Hodgkin’s lymphoma [32]. The increased lymphoma rates observed with anti-TNF therapy may reflect channeling bias, whereby patients with the highest risk of lymphoma preferentially receive anti-TNF therapy. Current data are insufficient to establish a causal relationship between anti-TNF and the development of lymphoma. It is also important to stress maintenance to improve outcomes and avoid/decrease antibody formation.

It has been shown that risk for squamous cell cancers increases with global immunosuppression [33]. A trend of elevated risk for cervical cancer with IBD and IBD medications was observed, but it was not statistically significant. Regular cervical cancer screening for women with IBD has been recommended [34]. It is especially important then, for patients on chronic immunosuppressive therapy to undero regular dermatologic examinations.

The FDA published a summary of 47 cases of heart failure (HF) associated with infliximab reported to the Adverse Events Response System (AERS) through January, 2002. In a patient who develops HF while on a TNF inhibitor, a drug-induced cause should be suspected and the medication should be discontinued. TNF inhibitors have rarely been associated with the development or exacerbation of neurologic disorders associated with demyelination, such as multiple sclerosis. However, the true nature of this association has not been established. Anti-TNF therapy should be discontinued immediately in any patient with suspected demyelination.

### Antibiotics

Mild perianal disease in Crohn’s may respond to antibioics. The most closely studied antibiotic for treatment of CD has been mertronidazole, this has effects similar to sulfasalaine and has superior efficacy as compared with placebo in mild to moderate disease [35]. Antibiotics are useful in UC in the setting of complications like pouchitis either alone or in combination with ciprofloxacin [36]. Other than this, there is no role for antibiotics in UC. Table 2 shows a list of major side effects of medications used for treatment of IBD.

**Table 2:** Major side effects of medicines used for treatment of IBD

Sulfasalazine and 5-ASA compounds	Hypersensitivity, sperm abnormalities, blood dyscrasias
Corticosteroids	Adrenal insufficiency, hyperglycemia, edema, osteonecrosis, cataracts myopathy, peptic ulcer disease, hypokalemia, osteoporosis, euphoria, psychosis, altered cell mediated immunity
Azathioprine/ Methotrexate	Blood dyscrasia, drug induced hepatitisand pancreatitis. AZA implied in T cell lymphoma, MTX in Hodgkin’s lymphoma
Metronidazole	Seizures, peripheral neuropathy, disulfiram reaction with alcohol
TNF–Alpha inhibitors	Anaphylaxis, superinfections, chest pain or rash, risk of reactivation of tuberculosis, rare occurrence of multifocal leucoencephalopathy

### Colon cancer surveillance

Are we waiting too long?

In an attempt to detect precancerous dysplasia and asymptomatic cancers in patients with IBD, the major gastroenterology societies recommend initiating colonoscopic surveillance beginning 8-10 years after the onset of disease in pancolitis and after 15-20 years for left-sided colitis, immediately and annually with primary sclerosing cholangitis (PSC) diagnosis.

There is data from Ullman et al which shows low grade dysplasia confirmed by a second expert IBD pathologist has high risk of progression and surveillance may not detect progression before metastatic spread. In a study presented by Lutgens and colleagues which identified patients diagnosed with IBD-associated colorectal cancer, of 104 patients diagnosed with IBD and colorectal cancer, 26% developed colorectal cancer before the start of surveillance. Although these data suggest that the diagnosis of colorectal cancer may be delayed or missed using current surveillance guidelines, it is not clear what the proper time point is for initiating surveillance, as every time point will reveal patients who develop cancer prior to the initiation of surveillance [37]. The internist and gastroenterologist taking care of the IBD patients should consider all the risk factors for colorectal cancer in ulcerative colitis including the duration and extent of colitis, PSC, family history of colon cancer, development of dysplasia, endoscopic appearance, and severity of inflammation at surveillance colonoscopy [38, 39].

Chromoendoscopy may eventually help to better define additional areas of biopsy. The use of chromoendoscopy for surveillance in IBD is not currently the standard of care; however, these studies add to a growing body of literature suggesting that this technique may improve the detection of dysplasia in IBD [40].

**Figure 1:**
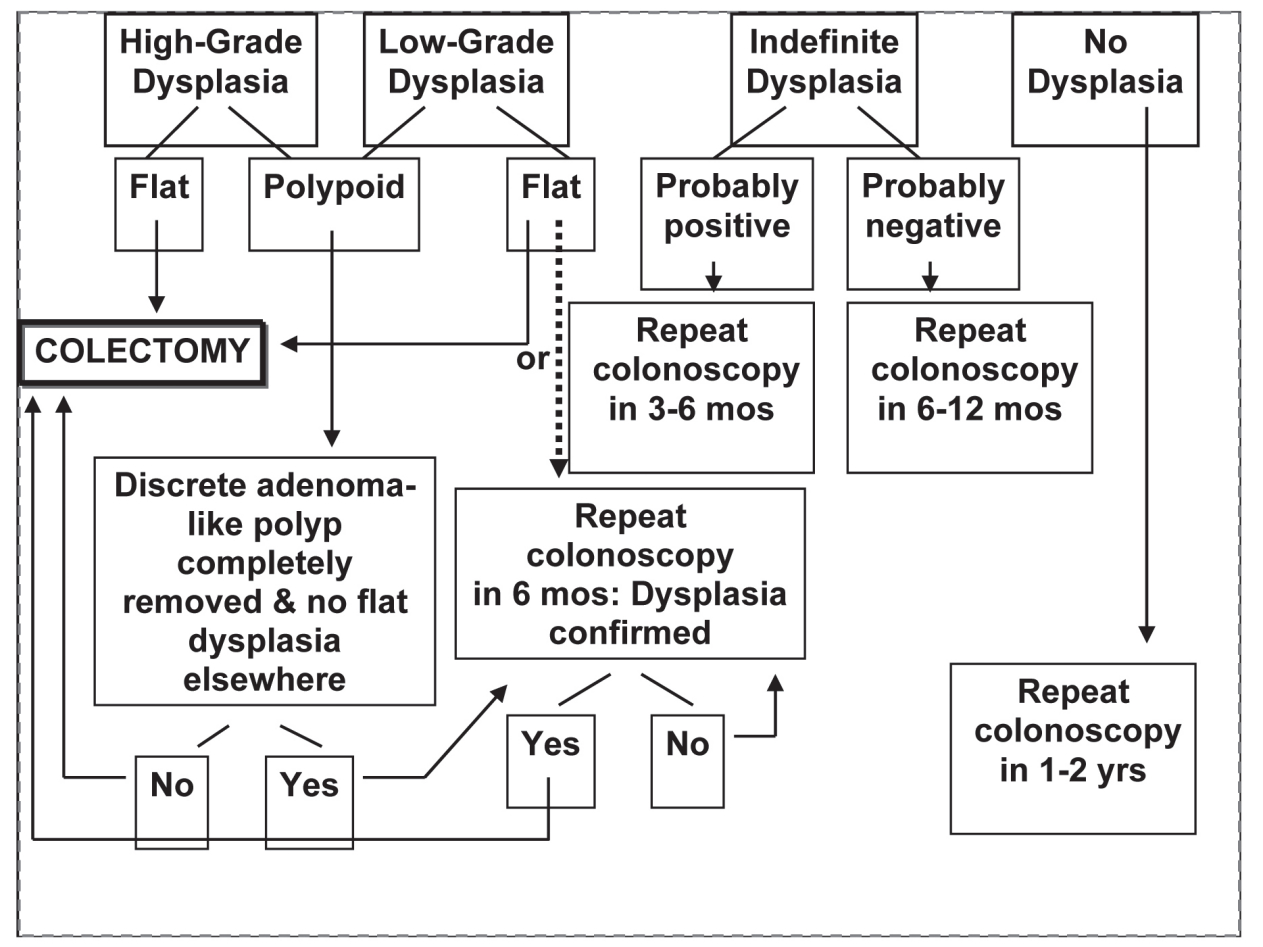
The suggested management of dyplasia in IBD

Colon cancer risk for Crohn's colitis is the same as UC [41]. The relative risk cancers of the small intestine is increased in Crohn’s, however small bowel cancers are rare with estimated ranges from 5.7- 7.5 cases per million [42]. In addition, a meta-analysis demonstrated an increased risk of small bowel, colon, extraintestinal cancers, and lymphoma in patients with CD [43]. Polypoid adenomas can be followed if complete polypectomy is performed and there is no dyplasia in adjacent flat mucosa. Figure 1 shows the suggested management of dyplasia.

The detection of precancerous dysplasia in IBD can be challenging because lesions can be flat, subtle, or difficult to detect on conventional surveillance colonoscopy [44]. Furthermore, few physicians take the required 32 biopsies in the colon needed to detect flat dysplasia [45]. Finally, meta-analysis of 5-ASA chemoprevention trials done by Rubin and Lashner shows a favorable role of this medicine in the prevention of cancer and dysplasia. The role of folate in chemoprevention has also been studies in murine models but needs to be confirmed in human intervention trials.

In a patient diagnosed with IBD, flare up is in the differential diagnosis of abdominal pain bouts (Table 3).

**Table 3:** Common causes of non- flare pain and diarrhea in IBD

1. Bile acid diarrhea
2. Increased NSAID use
3. Short gut syndrome
4.Infectious
5. Ischemic
6. Irritable bowel syndrome

### Toxic megacolon

It is important correctly identify and treat toxic megacolon. The diagnosis is based upon the history/physical and finding an enlarged dilated colon accompanied by severe systemic toxicity. The transverse or right colon is usually the most dilated, frequently greater than 6 cm and occasionally up to 15 cm on supine films. Repositioning of the patient results in redistribution of air in the colon and therefore location of air is not as important as the degree of dilatation. Anemia related to blood loss leukocytosis with a left shift and electrolyte disturbances are common and should be corrected.

All antimotility agents, opiates, and anticholinergics should be discontinued. High dose glucocorticoids should be initiated as soon as diagnosis is made and they do not increase the risk of perforation [46]. Broad spectrum antibiotics are started to minimize any septic complications. Parenteral nutrition is of limited value [47], Sulfasalazine and 5-ASA compounds have no role in patients with toxic megacolon due to IBD and should be initiated only after resolution of the attack.

Free perforation, massive hemorrhage, increasing transfusion requirements, worsening signs of toxicity, and progression of colonic dilatation are absolute indications for surgery. In addition, most surgical studies recommend colectomy if there is persistent colonic distention after 48 to 72 hour [48].

### Surgical treatment

Table 4 shows the indications for surgery in IBD. Surgical treatment is indicated in IBD if there is no response to optimal medical management and for dysplastic lesions. An honest discussion of the risks and benefits of the proposed operation with the patient and family is mandatory to avoid the frustrations stemming from unrealistic expectations. In patients who will receive an ostomy, a consultation with a stomal therapist is useful for selecting the ostomy site.

**Table 4:** Indications for surgery in IBD

Crohn’s disease	Ulcerative colitis
1. Intra-abdominal/ perianal abcess	1. Dysplasia complicating long standing UC
2. Complex fistulae	2. Recurrent, frequent relapses with poor quality of life despite optimal therapy
3. Mechanical complications like fibrotic strictures	3. Fulminant UC unreponsive to medical therapy
4. Fulminant CD unreponsive to medical therapy	

In order to provide adequate pre-operative care it is important to optimise the medical status, such as correcting anemia, addressing fluids/electrolytes. Most immunosuppressive therapy can be discontinued prior to sugery except glucocorticoids which should be tapered after surgery. Continuation of immunosuppressive therapy may be desirable in some patients with CD to prevent postoperative recurrence.

In UC, the usual procedures performed are proctocolectomy with ileostomy or colectomy with ileal pouch-anal canal anastomosis (IPAA). Segmental resections are sometimes performed for limited areas of Crohn's colitis but are inappropriate for patients with ulcerative colitis because of the risk of recurrent active inflammation or cancer developing in the remaining colon. Ileal pouch-anal canal anastomosis is usually avoided in patients with CD because of poorer functional outcomes and a higher failure rate.

The most frequent early complications are bowel obstruction, bleeding, pelvic and wound sepsis, transient urinary dysfunction, and dehydration due to high output from the ostomy. These can be addressed without re-operation. Late complications include stricture of the anastomosis, anal fistula and abscess, poor postoperative anorectal function, reduced fertility and pouchitis.

The only absolute contraindication to IPAA is anal sphincter dysfunction. Other contraindication includes suspected CD. The need for pelvic radiation also is a contraindication to pelvic reservoir construction. The diagnosis of UC must be certain before an ileal pouch reservoir is created in a patient with inflammatory bowel disease. It is important to avoid pouch surgery or total proctcolectomy for severe colitis. It may be important to discuss with the surgeon about avoiding total proctocolectomy for severe colitis and to leave all options open [49]. This is because of concerns for UC recurrence in pouch, infections of pouch, wound dehiscence made possible during a fulminant attack in which the patient's condition is compromised, with a potentially poor nutritional status, low albumin level, low hematocrit level, and complications of high-dose corticosteroid use.

CD is often complicated by fibrostenotic strictures that can be located within the whole gastrointestinal tract. Strictures can remain clinically asymptomatic over years until the intraluminal caliber causes obstruction. However, it is often difficult to differentiate between an inflammatory or fibrostenotic stricture. Ultrasound and MRI with the possibility to visualize mucosal blood flow are helpful in differential diagnosis. MR enterography is useful in distinguishing fixed strictures from inflammatory strictures at least in prelim studies.

Before initiating surgical interventions, many clinicians try at least one attempt of medical treatment for strictures suggested to have an inflammatory component. Corticosteroids are most commonly used in this clinical situation. Fibrostenotic strictures will not respond to medical therapy. Endoscopic ballon dilatations, stricturoplasty or resections are required [50], remembering that there is lack of data and long-term follow-up in endoscopic dilation of strictures and that malignancy may cause stricture.

Intra-abdominal abscess may need surgery even if it can be initially treated with percutaneous drainage with or without antibiotics. Patients who undergo surgical management are significantly less likely to develop a recurrent abscess compared to those who have been managed with antibiotics, 12 versus 56 percent [51].

Sutherland showed that the risk of reoperation 5 year after surgery in patients with CD was approximately 20% in nonsmokers and 36% in smokers. These figures increased to 41% and 70%, respectively, at 10 year. Female smokers and those with small bowel disease were at greatest risk. The effect of smoking can be reduced by long-term immunosuppression [52].

It is also important to identify the patients who are at high risk for recurrence of post-operative recurrence of CD. Even if macroscopically involved intestine is removed, the disease usually recurs proximal to, and at, the anastomosis. This often leads to the recurrent need for treatment of active disease, complications, and reoperation [53].

Patients with an ileocolic anastomosis are known to have higher recurrence rates than those with a colectomy and end-ileostomy [54]. Yet another predictor of recurrence is disease behavior. Repeat resection after the primary operation for perforating disease occurred in half the time, on average, compared to those with nonperforating disease. Time to first reoperation was 4.7 years in the perforating group compared with 8.8 years in the nonperforating [55].

It is important to identify clinical recurrence of the disease versus endoscopic recurrence. Rutgeerts and colleagues described endoscopic scoring instrument for clinical use in patients who underwent complete surgical resection, comparing endoscopic remission versus clinical remission versus the need for recurrent surgery. These researchers found that patients with grade III and IV endoscopic recurrence in the neoterminal ileum were at increased risk for a clinical recurrence. In high-risk patients, it would seem reasonable to implement prophylactic therapy using metronidazole initially later on immunomodulators. There is evidence that AZA is effective as a post-operative maintenance therapy though it is limited [25]. Landmark trial by Regueiro showed that administration of infliximab after intestinal resective surgery was effective at preventing endoscopic and histologic recurrence of CD.

In summary, there are many advances made in the management of IBD. With just a few precautions, health care providers could optimize the care and stall complications of the disease and its therapy.
